# Proof-of-Concept Digital-Physical Workflow for Clear Aligner Manufacturing

**DOI:** 10.3390/dj13100454

**Published:** 2025-10-02

**Authors:** Shih-Hao Huang, I-Chiang Chou, Mayur Jiyalal Prajapati, Yu-Hsiang Wang, Po-Kai Le, Cho-Pei Jiang

**Affiliations:** 1 Graduate Institute of Manufacturing Technology, National Taipei University of Technology, Taipei City 10608, Taiwan; jimmyhuang0321@gmail.com (S.-H.H.); t109300133@ntut.org.tw (Y.-H.W.); t109300132@ntut.org.tw (P.-K.L.); 2 Department of Stomatology, Yangming Branch, Taipei City Hospital, Taipei City 103212, Taiwan; joechou62@yahoo.com.tw; 3 Department of Dentistry, School of Dentistry, National Yang Ming Chiao Tung University, Taipei City 112304, Taiwan; 4 High-Value Biomaterials Research and Commercialization Center, National Taipei University of Technology, Taipei City 10608, Taiwan; mayurprajapati969@gmail.com; 5 Department of Mechanical Engineering, National Taipei University of Technology, Taipei City 10608, Taiwan

**Keywords:** additive manufacturing, dentistry, digital dental model, thermoforming, finite element analysis, Polyflow

## Abstract

**Background/Objectives**: Clear aligner therapy has become a mainstream alternative to fixed orthodontics due to its versatility. However, the variability in thermoforming and the limited validation of digital workflows remain major barriers to reproducibility and predictability. **Methods**: This study addresses that gap by presenting a proof-of-concept digital workflow for clear aligner manufacturing by integrating additive manufacturing (AM), thermoforming simulation, and finite element analysis (FEA). Dental models were 3D-printed and thermoformed under clinically relevant pressures (400 kPa positive and −90 kPa negative). **Results and Discussion**: Geometric accuracy was quantified using CloudCompare v2.13.0, showing that positive-pressure thermoforming reduced maximum deviations from 1.06 mm to 0.4 mm, with all deviations exceeding the expanded measurement uncertainty. Thickness simulations of PETG sheets (0.5 and 0.75 mm) showed good agreement with experimental values across seven validation points, with errors <10% and overlapping 95% confidence intervals. Stress analysis indicated that force transmission was localized at the aligner–attachment interface, consistent with expected orthodontic mechanics. **Conclusions**: By quantifying accuracy and mechanical behavior through numerical and experimental validation, this framework demonstrates how controlled thermoforming and simulation-guided design can enhance aligner consistency, reduce adjustments, and improve treatment predictability.

## 1. Introduction

Traditionally, the standard treatment method for correcting dental malocclusions has been the use of fixed orthodontic devices, such as metallic braces. Although very effective for severe cases, they come with several disadvantages, such as increased risk of plaque accumulation, tooth decay, and gingivitis, along with difficulties in brushing and flossing [1]. They can also lead to discomfort and irritation in the cheeks, lips, and tongue, leading to ulcers and soreness. Sometimes, patients are advised to avoid hard, sticky, or chewy foods to prevent wire displacement or bracket breakage [2,3]. These drawbacks of the traditional braces have driven the demand for a more discreet, comfortable, and digitally optimized option such as clear aligners [4,5]. Clear aligners have emerged as a preferred alternative to traditional fixed metal braces, owing to their comfort, esthetic appeal, and ease of maintenance [6,7]. The process of creating high-precision clear aligners to ensure effective correction has garnered significant attention.

Traditional aligners were manufactured using pre-digital methods, such as taking physical impressions of the jaws using plaster models or manual tooth setup, where teeth were trimmed and repositioned on a stone model [8]. A paradigm shift occurred with the digitization of the aligner manufacturing process, transforming it from a manual, labor-intensive process to a precise, scalable, and highly customized workflow. Moreover, the use of digital workflows enables more consistent aligner fit and force application, which are critical to achieving predictable tooth movement and avoiding treatment relapse [9]. From a clinical operations perspective, digital modeling also streamlines lab coordination, reduces turnaround time, and supports scalability in high-volume orthodontic practices [10,11].

The advancement of digital design and additive manufacturing (AM) technologies has significantly transformed the manufacturing of clear aligners. The introduction of intraoral scanners as a substitute for the conventional molds has improved accuracy, efficiency, and personalization of a patient’s dentition. The digital scans are then integrated into computer-aided design (CAD) software to virtually simulate treatment plans with high precision [12,13]. Also, these datasets can be used to facilitate the rapid fabrication of customized dental models through AM technologies such as material extrusion or vat photopolymerization [14,15,16]. The resulting 3D-printed models serve as physical substrates for producing clear aligners via the thermoforming process. Thermoforming is a manufacturing process in which a thermoplastic sheet is heated to become soft and pliable and then formed over a mold, such as a 3D-printed dental model. The forming process usually uses vacuum or pressure to form the shape and is then left to cool to retain its shape [17,18,19]. It is a cost-effective process to produce rapid and precise clear aligners over custom 3D-printed models. This streamlined workflow not only improves the fit and precision of aligners but also supports greater automation and digitalization across the orthodontic treatment pipeline. The advancement of this technology not only alleviates patients’ concerns about appearance but also enhances the overall treatment effect [20,21].

Although thermoforming is widely used to fabricate clear aligners, there are several challenges that affect its accuracy, fit, and mechanical performance. Issues like dimensional deformation due to non-uniform heating or pressure, causing variations in thickness across the aligners. This can lead to premature failure and cracks on the aligners, rendering them unsuitable for the treatment [22]. Despite the growing adoption of digital workflows, limited research has addressed deformation control and dimensional stability during the post-processing and thermoforming stages of clear aligner production. In particular, variables such as positive- or negative-pressure thermoforming and post-curing conditions can substantially affect the surface geometry of dental models and the accuracy of the resulting aligners [23].

Although clear aligner therapy has emerged as a popular alternative to fixed orthodontics, thermoforming of polymer sheets introduces geometric inaccuracies, thickness variations, and mechanical uncertainties that compromise aligner fit and force delivery. Thus, to simultaneously improve the effectiveness and efficiency of orthodontic treatment, this study employs digitally fabricated dental models using AM technology to conduct an analysis of forming errors, material deformation, and mechanical performance. The methodology integrates 3D deformation evaluation, thermoforming simulation using ANSYS Polyflow, and stress distribution analysis via finite element modeling. By simulating both manufacturing and in-use conditions, the study attempts to establish a data-driven foundation for process optimization, mechanical reliability, and improved clinical outcomes in clear aligner treatment. The developed digital-physical workflow capable of producing clinically accepted aligners with predictable force distribution can increase reliability and reduce manufacturing time.

## 2. Materials and Methods

### 2.1. Thermoforming Error Analysis

To analyze the thermoforming process, CloudCompare v2.13.0 (www.cloudcompare.org (accessed on 10 September 2025)) was used, which is an open-source 3D point cloud processing software [24]. It can evaluate the impact of various manufacturing processes, including post-curing and both positive- and negative-pressure thermoforming, on the accuracy and precision of digital dental models [25]. Point-to-point alignment was conducted using the Iterative Closest Point (ICP) algorithm, aligning the scanned post-processed models with the original STL design file. The ICP algorithm operates by identifying nearest-neighbor point pairs, calculating an optimal rigid body transformation (rotation and translation), and iteratively minimizing the distance until convergence or a predefined threshold is reached [26].

**Figure 1:**
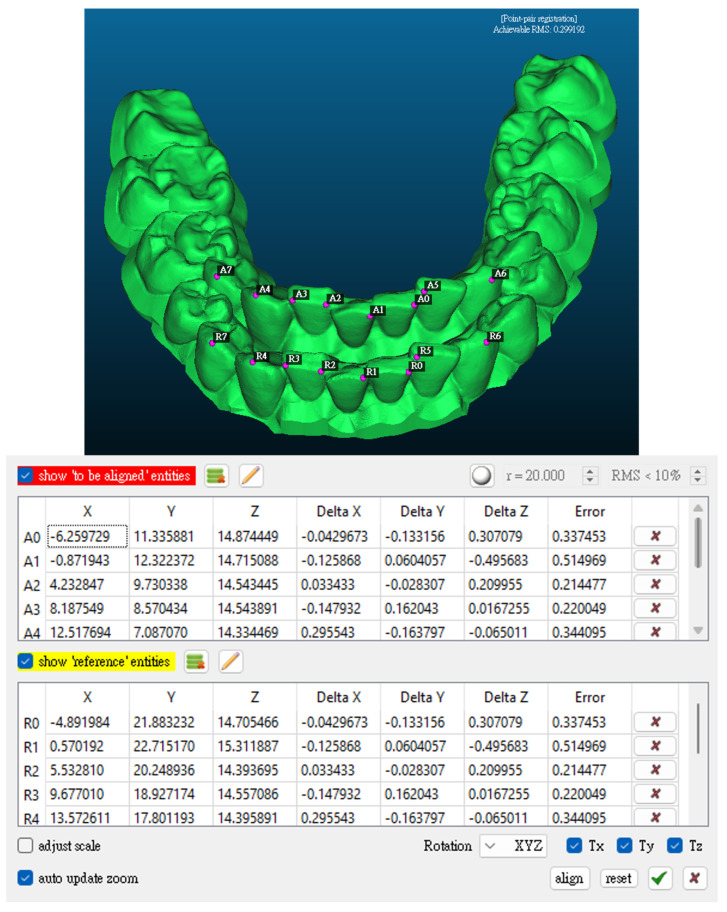
Comparison between the original design model and the scanned model.

**Figure 2:**
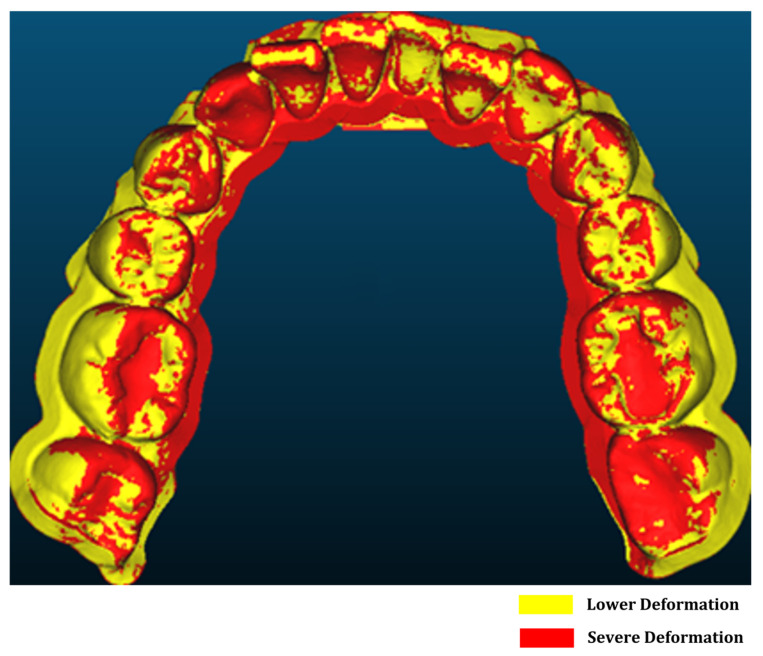
Visualization of deformation error results.

Once aligned, the cloud-to-mesh distance function in CloudCompare was used to calculate the shortest distance from each point on the scanned model to the corresponding reference mesh. This allowed for a quantitative assessment of geometric deviations introduced during post-processing and thermoforming. Figure 1 shows a direct comparison between the original design (model A) and the scanned thermoformed model (model B). The results were visualized as color-coded deviation maps, as shown in Figure 2. The red zones indicate regions of maximum deviation; that is, typically concentrated around the cusp tips and incisal edges. In contrast, yellow and green areas represent regions with lower deformation levels. These visualizations enabled a direct comparison of dimensional accuracy under positive- and negative-pressure thermoforming conditions and highlighted critical regions where surface precision is most affected. Deviation analysis was performed using a triangulated mesh with an average facet size of ~0.05 mm, consistent with the scanner’s native resolution, ensuring that discretization errors remained well below the observed deviations.

### 2.2. Materials, Thermoforming Parameters, and Measurement Methodology

Clear aligners are typically fabricated using thermoplastic materials such as polyethylene terephthalate (PET), polyurethane (PU), and various copolyesters. Among these, polyethylene terephthalate glycol-modified (PETG) is widely utilized due to its high optical clarity, favorable impact resistance, and excellent thermoformability [27,28]. In this study, PETG sheets (0.5 mm and 0.75 mm nominal thickness; density ≈ 1.28 g/cm^3^) manufactured by Scheu Dental GmbH (Am Burgberg, Iserlohn, Germany) were selected as the base material. Positive thermoforming was performed on Ministar S (Scheu-Dental GmbH, Am Burgberg, Iserlohn, Germany), whereas negative thermoforming was performed on Foremart S (MY YARD, Taoyuan, Taiwan). For positive thermoforming, PETG sheets were heated at a heater temperature of 220 °C for ~25 s, until a sag of 10–15 mm was observed. Forming was carried out under a positive pressure of 400 kPa (4 bar), maintained for 35 s, followed by air cooling under pressure until the sheet was stabilized (~60–90 s). For negative thermoforming, the heating was set to 130 °C for ~60 s, after which forming was performed under a vacuum level of −90 kPa (0.9 bar below the ambient), held for 35 s. Cooling was carried out under vacuum until the sheet reached room temperature (~60–90 s). In both cases, the pressure was controlled within ± 5 kPa. The selected forming pressures fall within the clinically relevant ranges for PETG aligner manufacturing, which are typically reported as 200–600 kPa for positive and −80 to −95 kPa for vacuum systems [28]. Ambient laboratory conditions during all forming steps were controlled at 25 °C and 50% relative humidity. For validation, three independent thermoformed aligner samples were fabricated using these processing conditions.

Three-dimensional printing was performed using a Phrozen Sonic Mega 4K (Phrozen Technology, Hsinchu City, Taiwan) DLP 3D printer. Printing was performed with a layer height of 0.05 mm, exposure time of 35 s for the base and 5 s for other layers, lifting speed of 60 mm/min, consistent with manufacturer-recommended parameters for dental resins. Models were post-cured under 405 nm UV light at an intensity of ~60 mW/cm^2^ for 15 min.

Thickness was measured using a digital thickness gauge with a resolution of 0.01 mm, calibrated before each session of measurement. Three independent aligner samples were measured, each at multiple points of the model. For each measurement point, mean thickness, standard deviation, and 95% confidence intervals were calculated. Surface deviations were assessed using an AROS Compact 3D scanner (GOM GmbH, Braunschweig, Germany), with a nominal resolution of 0.05 mm. Alignment between scanned and reference geometries was performed in CloudCompare using an iterative closest point (ICP) algorithm with a convergence tolerance of 0.001 mm, a maximum of 500 iterations, and random sampling of 20,000 points. The uncertainty budget for all the measurements is provided in Table S1.

### 2.3. Numerical Modeling of the Thermoforming Process

The thermoforming behavior of PETG was simulated using ANSYS Polyflow Student Version 2024 R1 (2600 Ansys Drive, Canonsburg, PA, USA). The dental model geometry was initially prepared and repaired in Meshmixer 3.5.0 (Autodesk, One Market Street, Suite 400, San Francisco, CA, USA) to ensure watertight integrity and proper surface continuity before being imported into Polyflow for meshing and boundary condition configuration. The Carreau–Yasuda Model in ANSYS Polyflow is a generalized Newtonian viscosity model used to describe fluids that exhibit shear-thinning behavior, transitioning from Newtonian to shear-thinning behavior. This model characterizes the material’s viscosity as a function of shear rate using the following equation:

(1) $$\eta \left(\dot{\gamma }\right)=\;\eta_{0}{\left[1+{\left(\lambda \dot{\gamma }\right)}^{a}\right]}^{\frac{n-1}{a}}\;$$

where is $\eta (\dot{\gamma )}$ the viscosity at the shear rate $\dot{\gamma }$, $\eta_{0}$ is the zero–shear viscosity, λ is the natural time (inverse of the shear rate at which the fluid changes from Newtonian to power-law behavior, a is the index that controls the transition from the Newtonian plateau to the power-law region, and n is the power law index. The coefficients of this equation were derived from the study conducted by Le et al., where they investigated the temperature-dependent viscosity of PETG, and were also listed in Table S2 [28].

**Figure 3:**
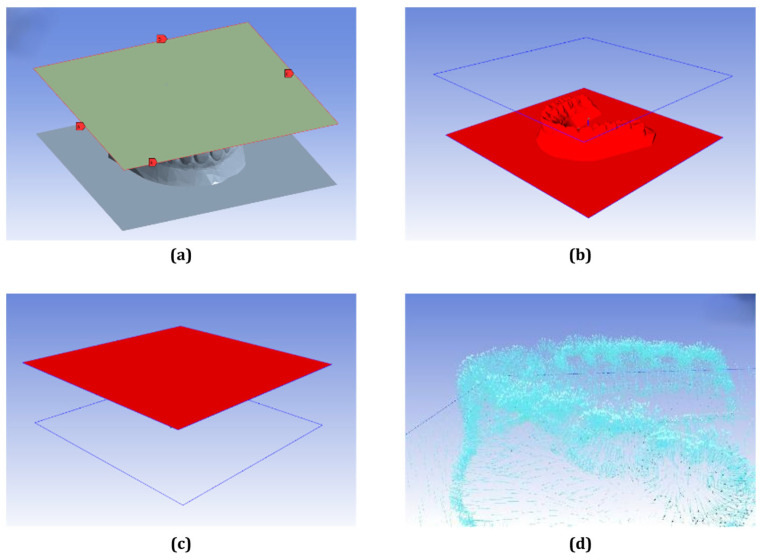
Polyflow simulation setup process: (**a**) pressure boundary assignment at PETG sheet edge, (**b**) mold geometry positioned beneath the thermoplastic sheet, (**c**) initial sheet and mold alignment before deformation, (**d**) deformation vector field visualizing material flow during forming.

In the simulation setup, the edges of the PETG sheet were defined as pressure boundaries (Figure 3a), while the mold and sheet geometries were assembled and oriented to ensure alignment between the pressure direction and mold surface normal (Figure 3b–d). The simulation was conducted to capture the deformation behavior and thickness changes in the PETG sheet during the thermoforming process. The resulting thickness distribution obtained from the simulation was validated against physical measurements taken from fabricated clear aligner samples. To evaluate the accuracy of the simulation, the relative error was calculated using the following equation:

(2) $$Relative\;Error\left(\%\right)=\frac{\left|Simulated-Measured\right|}{Measured}\times 100\%$$

This approach enabled a quantitative evaluation of simulation accuracy and model predictability by comparing the predicted thickness distribution with experimentally measured values from thermoformed aligners.

### 2.4. Setup for Stress Analysis During Wear

A static structural analysis using the Finite Element Method (FEM) module in ANSYS Workbench was conducted to evaluate the mechanical interaction between the clear aligner and the teeth during actual wear. The simulation was governed by the standard mechanical equilibrium equation:

(3) $$\left[K\right]\left\{u\right\}=\{F\}$$

where $\left[K\right]$ denotes the global stiffness matrix, $\left\{u\right\}\;$ represents the nodal displacement vector, and {F} is the external force vector. Solving this equation provides the nodal displacement field, which can subsequently be used to derive the resulting stress and strain distributions within the aligner and surrounding structures.

Note that this is an initial implementation; the tooth was modeled as a single dentin-like structure with fixed-base support. The periodontal ligament (PDL) and alveolar bone were not included, and loading was applied as displacement-driven constraints. This simplified setup was selected to demonstrate the feasibility of linking thermoforming simulations with wear simulations, rather than to reproduce exact intraoral biomechanics. Future refinements will incorporate multi-tissue tooth structures, the viscoelastic PDL, and force-controlled boundary conditions to improve physiological realism.

In the simulation, two materials were used to mimic real-world conditions: dentin represented the tooth structure and PETG was used to represent the clear aligner. Material properties are summarized in Table 1. Dentin, characterized by a high Young’s modulus and compressive strength, was selected to model the relatively rigid behavior of the tooth. In contrast, PETG, with its elastic properties, was suitable for capturing the aligner’s elastic deformation during wear, without imposing ultimate strength constraints in the simulation.

**Figure 4:**
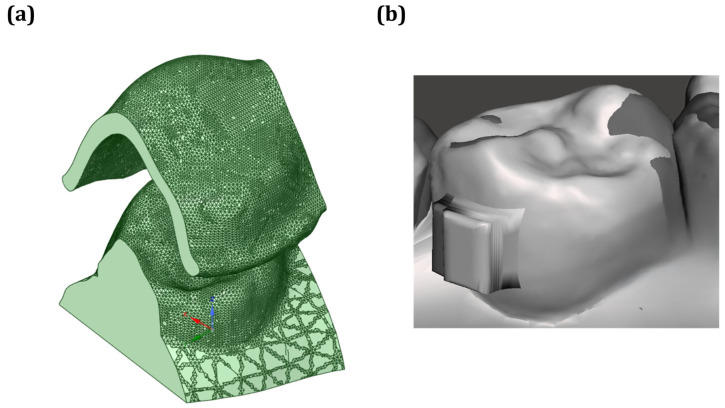
(**a**) Digital model of the aligner and dentition, and (**b**) contact model between the aligner and dentition for simulation analysis.

From Figure 4a, it can be seen that a localized simulation was carried out on a single premolar in order to strike a balance between the computational efficiency and the anatomical fidelity of the numerical simulation. The mandibular premolars were selected for testing as they serve as anchorage or movement teeth commonly used for aligner therapy. They have a relatively simple single-root morphology that facilitates finite element modeling [30,31]. The clinical treatment planning characteristics were used to determine the target tooth movement, which predominantly consisted of mesial translation in conjunction with rotation in the opposite direction of the standard clockwise direction (Figure 4b).

**Figure 5:**
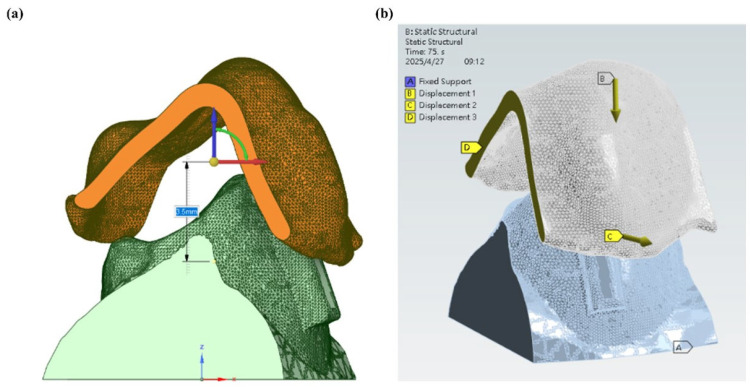
(**a**) Relative positioning of the aligner and dentition, and (**b**) simulation setup and boundary conditions.

In the simulation setup shown in Figure 5a, the aligner was initially positioned 3.5 mm above the tooth model to replicate the clinical insertion path. The base of the tooth was fully constrained to simulate gingival support, while a 3.5 mm displacement along the Z-axis (Displacement 1) was applied to the aligner to simulate seating onto the tooth. To prevent unintended movement during the simulation, displacement along the Y-axis was restricted (Displacement 2). Additionally, a compensatory displacement (Displacement 3) was introduced to avoid excessive interference between the aligner and the attachment block. The complete boundary condition configuration is shown in Figure 5b. The simplified single-tooth, displacement-driven model was developed to illustrate how thermoforming outputs can be integrated into stress analysis. The setup should therefore be interpreted as a conceptual demonstration rather than a clinically predictive simulation.

## 3. Results and Discussion

### 3.1. Forming Error Analysis Results

**Figure 6:**
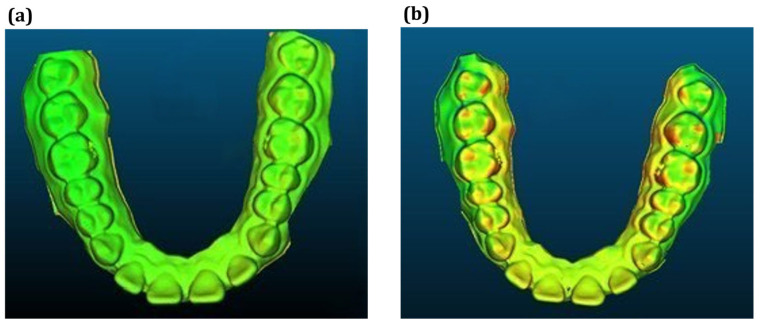
Deformation visualization under different thermoforming conditions: (**a**) thermoforming, (**b**) vacuum forming.

CloudCompare was used to visualize and quantify forming errors, enabling a comparative analysis of the effects of post-curing, positive-pressure thermoforming, and negative-pressure thermoforming on dental model deformation. As shown in Figure 6 and Table 2, all processes introduced measurable geometric deviations, with negative-pressure thermoforming resulting in the largest deformations, with deviations ranging from −0.477 mm to 1.059 mm [25,32,33].

In contrast, positive-pressure thermoforming provided superior surface control, demonstrating lower average and maximum deviation values (maximum ± 0.403 mm) [34]. These findings highlight the exceptional surface conformality and stability achieved with positive-pressure forming. This is likely due to the uniform pressure distribution during material shaping. This distinction between the negative- and the positive-pressure thermoforming is crucial in clinical treatments. Misfitting aligners, resulting from excessive deformation, can lead to ineffective force transmission, discomfort, and prolonged treatment duration. Therefore, positive thermoforming not only enhances geometric fidelity but also directly impacts treatment predictability and patient satisfaction.

Additionally, the comparison between the pre- and post-curing stages revealed that the post-curing stress relaxation contributes to minor deviations. This step can affect the aligner fit and must be considered during model preparation. A summary comparison of the deformation effects from various processing stages is presented in Table 2 and visually represented in Figure 6. The deviations between the models ranged from 0.12 to 1.06 across all the comparison stages. Since these values exceed the expanded measurement uncertainty (±0.06 mm, 95% CI), the observed deviations represent true differences between processing stages rather than measurement artifacts. These results underscore the importance of controlling post-processing variables to minimize forming errors and ensure consistent aligner quality.

### 3.2. Thermoforming Simulation Results

**Figure 7:**
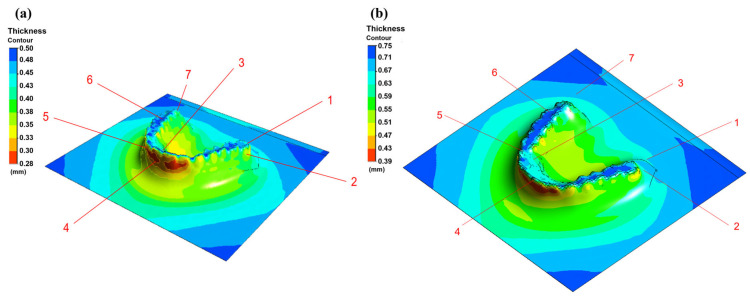
Simulated thickness distribution of (**a**) 0.5 mm and (**b**) 0.75 mm PETG sheet during thermoforming (numbers represent the zones for thickness measurements).

**Figure 8:**
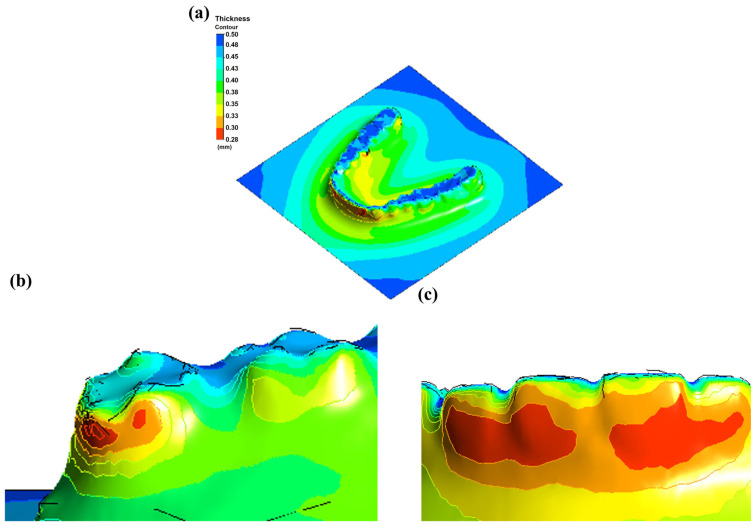
Thermoforming simulation results for 0.5 mm PETG sheet: (**a**) overall thickness distribution, (**b**) wisdom tooth region, (**c**) incisor region.

Simulation results for PETG sheets with thicknesses of 0.75 mm and 0.5 mm are presented in Figure 7 and Figure 8, illustrating the thickness distribution and variation patterns across different regions of the aligner during thermoforming. For the 0.75 mm PETG sheet, although some degree of thinning occurred, the overall thickness distribution remained relatively uniform. Critical regions, including the central incisors and molars, retained thicknesses above 0.6 mm, while the thinnest areas remained above 0.45 mm. These results indicate that using thicker PETG sheets helps maintain structural integrity, reducing the risk of material failure, such as tearing or excessive deformation during the forming process.

In contrast, the 0.5 mm PETG sheet exhibited more pronounced thinning during the thermoforming process. Notably, regions with higher surface curvature, such as the anterior incisors and distal molars, showed thickness reduction, with values dropping below 0.3 mm. This degree of thinning may compromise the mechanical strength and clinical durability of the aligner, underscoring the importance of both material selection and process parameter optimization to ensure consistent performance and patient safety.

**Figure 9:**
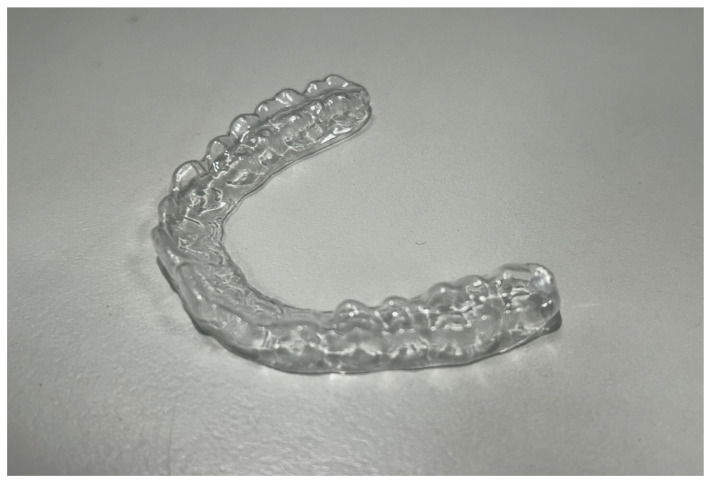
Thermoformed aligner sample.

**Figure 10:**
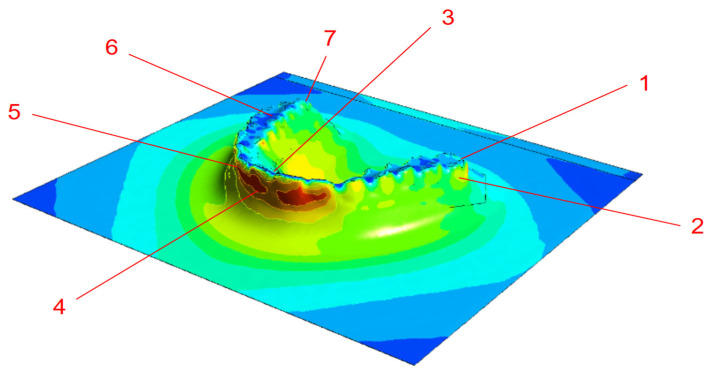
Thickness measurement points.

To evaluate the accuracy of the simulation, 0.5 mm and 0.75 mm thick PETG sheets were used to fabricate aligners via thermoforming and experimentally measured, as shown in Figure 9. Thickness measurements were taken in selected regions that corresponded to high-deformation zones identified in the simulation (Figure 10). The comparison of measured and simulated thickness values for 0.5 mm and 0.75 mm PETG sheets is presented in Table 3, reported as mean ± SD with 95% confidence intervals to account for measurement uncertainty. The overlapping CIs indicate that the simulations agree well with the experimental data within the limits of variability. These results are presented as preliminary feasibility-level findings. Formal hypothesis testing was not performed, and the conclusions are therefore framed as indicative trends rather than definitive statistical outcomes [17,35].

These results suggest a strong dependency of the mechanical integrity of the aligner on both the material thickness and geometric complexity. While using thicker sheets may increase comfort concerns or reduce flexibility, they offer improved durability and reduce the risk of fractures during usage. Therefore, a careful balance must be made between the structural robustness and patients’ comfort. This is where guided simulation tools assist during the product design.

In principle, the simulation framework presented here can be used in dental labs or clinics to virtually test and refine aligner designs before fabrication. This will minimize trial and error and reduce material and energy wastage. Additionally, it enables automated adjustment of the design parameters for patients with complex dental anatomy.

The present validation relies on seven measurement points, which provide limited spatial coverage and may not fully capture errors in complex geometries. These findings should therefore be regarded as preliminary. Future work will adopt systematically stratified sampling with more points to achieve spatial coverage and statistical rigor.

### 3.3. Stress Analysis During Wear

**Figure 11:**
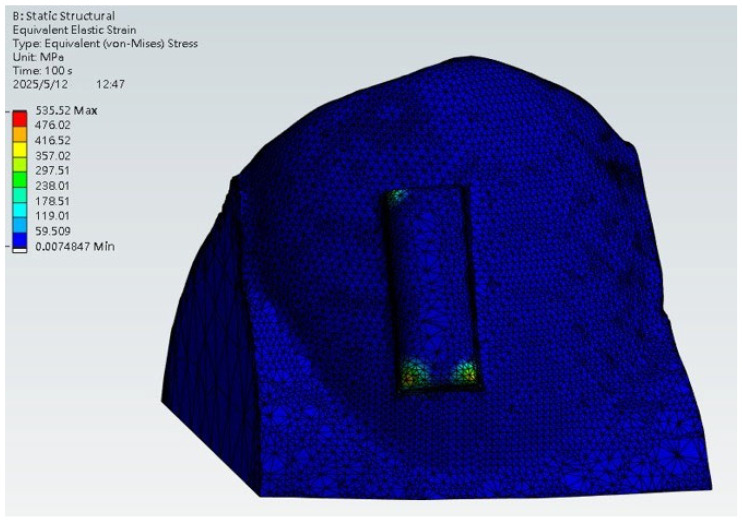
Equivalent stress distribution of the aligner during wear.

The finite element analysis results illustrating a clear aligner stress distribution are presented in Figure 11. The highest stress concentrations were observed at the contact interfaces between the aligner and the attachment block, highlighting the primary zones of force transmission. In the simulation, the base of the tooth was fixed to replicate gingival support, and the tooth structure was modeled using dentin properties to approximate clinical stiffness. This observation aligns with the expected clinical mechanism of the tooth movement.

It is important to note that this model represents a conceptual first-step numerical simulation. By omitting the PDL periodontal ligament (PDL) and alveolar bone and treating the tooth as a quasi-rigid structure, the analysis is likely to overestimate stiffness and local stress magnitudes. Similarly, the use of displacement-driven loading may not fully capture the force-controlled nature of clinical aligner engagement. Thus, the stress values reported here should be interpreted as illustrative of contact and force-transfer patterns rather than as a prediction of exact intraoral stresses. The observed stress concentrations at attachment–aligner interfaces are nevertheless consistent with clinically expected force delivery zones. This demonstrates the utility of the workflow for identifying relevant mechanical interactions, while providing a foundation for future models that will incorporate multi-tissue anatomy and realistic loading conditions.

However, it is important to note that the model omits physiological structures, which likely results in an overestimation of stiffness and localized stress magnitudes. As a consequence, the simulated displacement and stress values may deviate from actual intraoral conditions. Additionally, treating the tooth as a quasi-rigid body may lead to an overprediction of contact stress. To improve biological fidelity and clinical relevance, future studies should incorporate full anatomical modeling that includes PDL elasticity and alveolar bone support.

Despite these limitations, the simulation effectively demonstrates the aligner’s force delivery mechanism and contact behavior, providing a valuable foundation for future optimization of aligner design and force prediction strategies. In practice, it offers clinicians a tool to simulate aligner–tooth interaction under various design conditions, guiding decisions on force direction, aligner fit, and pressure zones.

### 3.4. Comparison Between Traditional and Proposed Workflow

The transition from conventional to fully digital workflows in clear aligner manufacturing presents advantages in terms of accuracy, repeatability, and clinical efficiency. The proposed workflow in this study integrates intraoral scanning, AM, thermoforming simulations, and FEA to create a highly precise data-driven modeling process. The integration of AM allows for a rapid and customizable model fabrication, eliminating the material inconsistencies of the plaster-based traditional workflows. Table 4 shows the comparison of the two processes.

Thus, digital workflow improves the engineering robustness of the aligners and also aligns with the evolving trend towards automation and personalized care in orthodontics. These features enable digital workflow to promote large-scale orthodontic device manufacturing and direct-to-consumer aligner manufacturing, where speed, consistency, and data integration are crucial for market competitiveness. As the field of orthodontics evolves, combining digital workflow with emerging technologies such as AI-driven design automation and intraoral sensor feedback may further transform orthodontic treatment into a predictive and adaptive process, ultimately improving both patient and practice efficiency. We acknowledge that the present feasibility study did not include randomization protocols or repeated temporal stability testing. These steps will be incorporated in future work to further strengthen process control. Moreover, when considered alongside existing evidence, these results highlight an opportunity to align digital–physical workflows in clear aligner manufacturing with other emerging technologies. For example, integration with smartphone applications has been shown to enhance patient engagement and clinical data collection in dentistry, while AI is increasingly being applied to improve diagnostic reliability [40,41]. Incorporating such tools into the digital orthodontic workflow could further improve the robustness, efficiency, and clinical applicability of the aligner fabrication process.

From a clinical perspective, this study demonstrates the feasibility of incorporating digital simulation into aligner fabrication workflows to improve the predictability of fit and force distribution. Clinicians may interpret this workflow as an early framework for improving aligner reproducibility, but further research with larger datasets and patient-level validation is required before clinical implementation.

## 4. Conclusions

This work demonstrates a feasibility-level digital workflow combining AM, thermoforming simulation, and FEA to analyze aligner accuracy and mechanics. The following are the concluding remarks from this study:

- Positive-thermoforming achieved superior fidelity, reducing geometric deviations to 0.3–0.4 mm compared with 0.48–1.06 mm under negative pressure.
- PETG sheet thickness simulations correlated well with experimental measurements with deviations within 10% and supported by 95% confidence intervals, confirming predictive potential.
- Stress analysis showed concentrated load transfer at aligner–attachment interfaces, aligning with clinically expected force delivery patterns, though the simplified model (single tooth, displacement driven) should be interpreted as illustrative.
- Overall, the workflow highlights how numerical simulation and controlled processing can improve structural consistency and predictability in aligner manufacturing.

Future studies can be conducted to incorporate temperature-dependent rheology, multi-tooth anatomies, force-controlled loading, and expanded measurement datasets to strengthen clinical translation.

## Figures and Tables

**Table 1:** Material properties for stress analysis [22,29].

Property	PETG Sheet	Tooth Tissue
Density	1.28 g/cm^3^	2.1 g/cm^3^
Young’s Modulus	2 GPa	18 GPa
Poisson’s Ratio	0.33	0.3
Ultimate Tensile Strength	–	105 MPa
Ultimate Compressive Strength	–	300 MPa

**Table 2:** Comparison of deviation ranges under different process conditions.

Comparison Stage	Deviation Range (mm)
Original vs. Post-cured model	−0.12~0.14
Post-cured vs. Positive-pressure model	−0.19~0.24
Post-cured vs. Negative-pressure model	−0.33~0.56
Original vs. Positive-pressure model	−0.30~0.40
Original vs. Negative-pressure model	−0.48~1.06

**Table 3:** Comparison of measured and simulated thickness data for PETG sheet.

	0.05 mm				0.75 mm			
Point	Measured (mm)	95% CI (mm)	Simulated (mm)	Error (%)	Measured (mm)	95% CI (mm)	Simulated (mm)	Error (%)
1	0.42 ± 0.02	0.37–0.47	0.43	−2%	0.71 ± 0.05	0.59–0.83	0.73	3%
2	0.35 ± 0.04	0.25–0.45	0.35	0%	0.58 ± 0.02	0.53–0.63	0.54	7%
3	0.43 ± 0.07	0.26–0.6	0.41	4%	0.71 ± 0.02	0.66–0.81	0.70	4%
4	0.32 ± 0.05	0.2–0.44	0.31	3%	0.48 ± 0.04	0.38–0.58	0.47	2%
5	0.32 ± 0.02	0.27–0.37	0.31	3%	0.42± 0.07	0.24–0.58	0.47	8%
6	0.33 ± 0.07	0.16–0.5	0.31	6%	0.7± 0.03	0.55–0.85	0.73	4%
7	0.47 ± 0.03	0.4–0.55	0.49	−4%	0.64± 0.02	0.59–0.69	0.66	3%

**Table 4:** Comparison between the traditional methodology and digital design workflow.

Parameters	Traditional Methodology	Digital Design Workflow	Reference
Accuracy	Prone to manual error and material inaccuracies, such as thickness variations and thinning.	Direct printing enables uniform thickness distribution and improved accuracy in gap widths.	[28,36]
Repeatability	Manual trimming and heating cycles can lead to variability.	Digital CAD/CAM files ensure reproducibility and allow automated pre-fabrication quality checks.	[6,37]
Fabrication time	Involves multiple steps (printing, thermoforming, trimming)	Eliminates physical modeling, printing, and post-processing, reducing overall time.	[38,39]
Customization	Manual process limits customization	Full digital customization via CAD modeling.	[28,39]
Clinical Predictability	Poor fitting and variability are force delivery is predominant.	Allows more personalized biomechanics and potentially more predictable outcomes once validated.	[38,39]

## Data Availability

The data that support the findings of this study are available from the corresponding author upon reasonable request.

## Supplementary Materials

The following supporting information can be downloaded at: https://www.mdpi.com/article/10.3390/dj13100454/s1, Table S1: Uncertainty Budget, Table S2: Carreau-Yasuda Parameters [28].
