# Supplementary material for: Proof-of-Concept Digital-Physical Workflow for Clear Aligner Manufacturing
Source: Dent J (Basel). 2025 Oct 2;13(10):454. doi: 10.3390/dj13100454 (PMC12563393; doi:10.3390/dj13100454)
Supplement: Supplementary file 1 [file dentistry-13-00454-s001.zip › dentistry-3828717-supplementary.pdf]

Supplementary Information

Table S1: Uncertainty Budget

Source of Uncertainty	Contribution	Notes
Point Gauge resolution	± 0.01 mm	Manufacturer specification
ATOS Compact 3D Scanner	± 0.05 mm	Nominal resolution
ICP alignment tolerance	± 0.001 mm	Convergence criterion
Operator repeatability	± 0.02 mm	Based on 3 samples
Total expanded uncertainty (95% CI)	± 0.06 mm	Combined estimate

Table S2: Carreau-Yasuda Parameters [28]

Temperature (C)	η_0 $(Pa.s)$	λ (s)	a	n
130	1,043,395	1.2363	0.56	0.09
150	135,215	0.1682	0.60	0.10
170	26,704	0.0481	0.71	0.18
190	8346	0.0207	0.81	0.26
210	3548	0.0131	0.90	0.35
230	1627	0.0117	1.20	0.52
